# Case Report: Transient myeloproliferative disorder with trisomy 21 in blast cells

**DOI:** 10.3389/fped.2025.1604803

**Published:** 2025-05-30

**Authors:** Aleksandra Zacny, Barbara Saniewska, Maria Orzeł, Beata Borek-Dzięcioł, Bożena Kociszewska-Najman

**Affiliations:** ^1^ Student Scientific Association ProNeo at the Department of Neonatology and Rare Diseases, Medical University of Warsaw, Warsaw, Poland; ^2^ Department of Neonatology and Rare Diseases, Medical University of Warsaw, Warsaw, Poland

**Keywords:** newborn, transient myeloproliferative syndrome, blast cells, leukemia, down syndrome, trisomy 21, case report

## Abstract

**Background:**

Transient myeloproliferative disorder is a clonal myeloproliferative syndrome that occurs in the presence of mutations in the GATA1 gene and chromosome 21 trisomy. It affects almost exclusively newborns with Down syndrome and usually resolves spontaneously. Neonatal leukemia is a rare childhood disease. Its prognosis is worse. We report a novel case of transient myeloproliferative disorder in a neonate without phenotypic features of Down syndrome, emphasizing the importance of comprehensive genetic diagnostics in atypical presentations.

**Case presentation:**

We present a case of a 4-day-old female neonate without phenotypic features of Down syndrome with suspected proliferative hematopoietic disease. A blood smear at birth showed severe anemia, leukocytosis and the presence of blasts. Abdominal ultrasound showed hepatosplenomegaly. In the bone marrow, 70.2% blast cell infiltration was described. An abnormal karyotype of 47XX+21 and GATA1 mutation were detected only in the blood cells. Transient myeloproliferative syndrome with t21 mosaicism was diagnosed. The patient received cytoreductive treatment according to the AML BFM protocol.

**Conclusions:**

This case highlights the importance of genetic testing in neonates with congenital anemia and hyperleukocytosis, particularly when Down syndrome is not phenotypically apparent. Detecting trisomy 21 mosaicism and the GATA1 mutation is critical for diagnosing transient myeloproliferative disorder, planning the best treatment and determining prognosis.

## Introduction

Transient myeloproliferative disorder (TMD), known as transient leukemia or leukemoid reaction, is a clonal myeloproliferative syndrome. The blood count shows leukocytosis, thrombocytopenia and a variable blast count. Anemia is not always present. Symptoms suggestive of TMD include hepatosplenomegaly, cardiac and pancreatic changes and prolonged jaundice (1).

TMD is considered to occur in the presence of mutations in the GATA1 gene and trisomy of chromosome 21 (t21). The disease almost exclusively affects infants with Down syndrome (DS) and tends to resolve spontaneously (1, 2). However, some patients develop a severe form of the disease and die in early childhood. After a period of remission, approximately 20% of children develop acute myeloid leukemia (AML) associated with DS (3).

Neonatal leukemia, occurring up to 28 days of age, is one of the rarest childhood diseases. The most common form is AML. Laboratory findings include leukocytosis, anemia and thrombocytopenia. However, the prognosis of patients with leukemia is significantly worse than in patients with TMD (4).

The authors describe a case of a neonate without phenotypic features of t21, in whom the suspicion of a proliferative blood disease was raised based on the symptoms. In the absence of DS features, neonatal leukemia was originally suspected. Subsequent diagnosis allowed the diagnosis of t21 mosaicism with the abnormal karyotype presented only in blood cells, which changed the initial prognosis. Based on these findings, the final diagnosis was changed to TMD, appropriate treatment was started.

## Case description

A female neonate, from pregnancy 3 delivery 1, was admitted to the hospital on the 4th day of life with suspected proliferative hematopoietic disease. The baby was born at 30 weeks of gestation by caesarean section due to threatening asphyxia. Birth weight was 1,600 g. At birth, the condition of the neonate was severe, the baby got 2/6/6/6 points on the Apgar score. Umbilical cord blood gases showed a blood pH of 7.12. The pregnancy was complicated by gestational diabetes mellitus type 1. 4 days before delivery a suspicion of cardiomegaly and fetal anemia was raised. Clear amniotic fluid drained during caesarean section.

After birth, the baby required resuscitation, intubation, mechanical ventilation and surfactant administration. Laboratory tests after birth showed severe anemia: hemoglobin 5.4 g/dl, hematocrit 15.5%. Irradiated leukocyte-depleted red blood cell concentrate was transfused. In addition, the blood count test showed leukocytosis of 124–128,000/µl and a blood smear showed 44% blast cells.

On admission to the Neonatal Intensive Care Unit, the patient's condition remained severe with respiratory and circulatory failure. A supply of catecholamines was administered until day 4 and respiratory support was provided until day 22. After birth, empirical antibiotic therapy was instituted, initially ampicillin with gentamicin. From day 9, after the diagnosis of pneumonia and in view of persistent hyperleukocytosis and an increase of C-reactive protein (CRP), piperacillin with clavulanic acid was started. Antibiotic therapy was continued for 14 days until resolution of the inflammatory lung lesions, normalization of CRP and negative blood cultures. During the infection, platelet concentrate was transfused once due to thrombocytopenia (30,000). Follow-up platelet values on subsequent days of hospitalization remained within normal limits. On physical examination, there were no abnormalities, except for hepatomegaly confirmed by ultrasound. Skin without petechiae or other changes.

An increase in leukocytosis to a maximum of 162,000/µl was observed, with a normal platelet count. Peripheral blood smear detected 80% blast cells. Biochemical tests showed acidosis and hyperkaliemia. Phosphates, calcium and uric acid remained within the range of norm.

A bone marrow biopsy was performed - the microscopic examination showed an infiltration of blast cells, representing 70.2% of the bone marrow tissue. Cytoreductive treatment with cytarabine was initiated on the 5th day of life due to suspected congenital acute leukemia with hyperleukocytosis. The patient received the treatment according to the AML BFM 2019 protocol for 5 days (day 1 - 1 mg, day 2 - 1 mg, day 3 - 1 mg, day 4 - 2 mg, day 5 - 2 mg). The response to treatment was good, and the rate of leukocytosis reduction was safe. Prophylaxis of tumor lysis syndrome with rasburicase was used (0.3 mg once a day for 3 days). In the control microscopic peripheral blood smear, no blast cells were observed.

A cytogenetic study showed trisomy 21 in the peripheral blood cells. Regarding the suspicion of DS in a neonate with hyperleukocytosis and hepatosplenomegaly, a suspicion of TMD was raised. A buccal mucosal swab was taken to differentiate the germinal from the somatic lesion and to assess the presence of t21 in other cells. Based on the results, the presence of T21 was ruled out in the general cell population; trisomy 21 was detected exclusively in the blast cells.

However, Sanger sequencing revealed the presence of a heterozygous c49dupC (pGln17ProfsTer23) variant in exone 2 of the GATA1 gene, which is characteristic of TMD. This duplication causes a frameshift and leads to a premature termination of translation.

In view of the above findings, the girl was diagnosed with TMD with the presence of t21 in blast cells.

The mother's previous pregnancies ended in miscarriage. No genetic testing was performed on the fetal tissue. The parents have undergone genetic evaluation and the same mutation was found in the mother's blood cells.

On day 39, due to chemotherapeutic treatment and suspected in the ultrasound examination microabscesses of fungal etiology in the liver, treatment with amphotericin B was initiated. In the follow-up, the liver lesions gradually resolved and blood and urine cultures did not show any fungi. Subsequent ultrasound examinations showed a decrease in liver size, with persistence of hyperechoic changes. After 6 weeks of antifungal treatment, therapy was discontinued. The liver lesions were considered to be persistent and not consistent with active fungal infection.

During the extensive diagnostics, congenital immune defects were ruled out. Results of virological tests (EBV, CMV, HHV6, HSV1, HSV2) were also negative. The cerebrospinal fluid culture remained sterile and the neurological panel was negative. Echocardiography did not confirm the prenatally observed cardiomegaly. The girl developed harmoniously, in accordance with her postconceptional age.

During hospitalization, due to anemia, the girl received 10 irradiated red blood cell concentrates. The girl remained under constant oncological supervision, and on day 60, she was transferred to the Hematology Department for further treatment. On the 77th day of life, the girl was discharged home in good general condition with a recommendation for follow-up in the Hematology Department.

**Figure 1:**
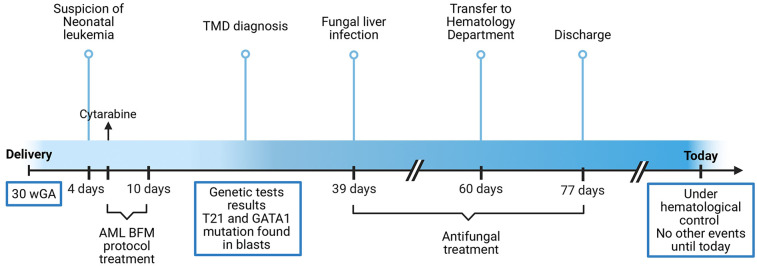
Timeline of the events. Abbreviations: WGA, weeks of gestational age; TMD, transient myeloproliferative disorder; T21, trisomy 21.

A follow-up visit at 6 months of age showed no relapse. Normal cognitive and neurological development was noted. The events presented in the case report may be found in Figure 1.

## Discussion

The detection of blasts in the blood of the patient posed 3 possible scenarios: congenital leukemia, t21 mosaicism with TMD and DS with TMD.

Neonatal leukemia is a congenital disease or one that occurs up to 28 days of age. It most commonly presents as AML, acute lymphoblastic leukemia or a mixed form. The characteristic symptoms of leukemia in the newborn are hepatosplenomegaly and skin lesions. In about half of the cases, there is central nervous system involvement. Blood counts show leukocytosis, anemia and thrombocytopenia. Infants with leukocytosis may develop respiratory, renal or cardiac failure. In most cases, patients require intensive multi-drug chemotherapy. Unfortunately, this is a treatment with a narrow therapeutic index that does not guarantee long-term remission (4).

In the described patient, anemia, leukocytosis and hepatomegaly were observed from birth. When the presence of blasts in the blood was detected, acute congenital leukemia was suspected. Cytoreductive treatment was implemented and genetic testing was performed. The result showed the presence of t21 in the peripheral blood cells. Based on this, neonatal leukemia was ruled out and DS with TMD was suspected.

T21 resulting in the development of DS is the most common chromosomal aberration worldwide. Patients present with a set of characteristic phenotypic features: dysmorphia, developmental delay, congenital heart defects, hearing and vision impairment. The disease is also associated with an increased incidence of leukemia and Alzheimer's disease. Up to 30% of newborns with DS will develop TMD (5). Unlike congenital leukemia, TMD is characterized by spontaneous remission in most cases. TMD rarely presents with skin lesions, which are present in most patients with congenital leukemia. There is usually no anemia, but megakaryocytes may be present in the blood and platelet counts may vary (4). Characteristic clinical features of TMD are hepatosplenomegaly, liver failure, coagulation abnormalities and pericardial effusion (6).

The described patient had no phenotypic features of DS. A buccal mucosal swab was taken to differentiate the somatic from the germinal lesion. The result ruled out the presence of t21 in the examined material. The detection of blasts in the blood of patients without DS characteristics may suggest the presence of t21 mosaicism. In such patients, cytogenetic analysis is indicated. This test can determine in which cells the mutation is present. T21 may be present in blood cells, fibroblasts or lymphocytes T. It is also possible that t21 is present only in a specific cell population, as in our patient (7). In the molecular study carried out, a mutation in the gene encoding the transcription factor GATA1 was found. The mutation of this gene is characteristic of blast cells found in the blood of patients with TMD. The abnormal GATA1 protein does not properly regulate the final stage of megakaryocyte differentiation. This results in the accumulation of their precursors. Detection of this mutation, together with t21, is fundamental to the diagnosis of TMD (3). There were no cases reported with the same mutation variant, however, many other frameshift mutations resulting in early termination of translation were found and these were also associated with TMD (8).

In the case described by Prudovsky et al., the female newborn presented with a similar mutation (a duplication in exon 2 causing a frameshift and premature stop codon) and t21 mosaicism. Despite similar initial laboratory results, the child experienced spontaneous remission by the 16th day of life and required fewer interventions than described patient (6). In contrast, the case reported by Brandie et al. involved a more extensive duplication of the *GATA1* gene and resulted in stillbirth of a male fetus, suggesting a more severe phenotype (9). This may be due to the *GATA1* gene being located on the X chromosome.

Most children with TMD will not require treatment due to the spontaneous remission of the disease. Current recommended therapies for the life-threatening form emphasize cytoreduction. However, in some patients, it is necessary to implement supportive treatment, which includes steroid therapy and exchange transfusion. Low doses of cytarabine are often effective in lowering blast levels (3). T21 mosaicism may affect the development of other diseases in the future. It is suspected that this mutation may predispose to the development of immune disorders, type 1 diabetes and asthma. Chromosome 21 mosaicism is associated with an increased risk of developing Alzheimer's disease at a young age (10).

## Conclusions

Any severe disease in a newborn is life-threatening. Congenital anemia and hyperleukocytosis can be indicative of proliferative diseases, necessitating extensive diagnostics, including genetic testing. The presence or absence of chromosomal abnormalities guides the course of treatment for the leukemic reaction. Detection of the TMD-specific GATA1 marker enables optimal treatment planning and helps determine the prognosis.

## Data Availability

The original contributions presented in the study are included in the article/Supplementary Material, further inquiries can be directed to the corresponding author.
